# Presentation of glomerulocystic disease in a young onset diabetes: A case report

**DOI:** 10.1097/MD.0000000000036952

**Published:** 2024-01-26

**Authors:** Wen-Hung Huang, Kun-Hua Tu, Tai-Di Chen, Cheng-Hao Weng, Ching-Wei Hsu

**Affiliations:** a Department of Nephrology, Clinical Poison Center, Chang Gung Memorial Hospital, Linkou, Taiwan; b Chang Gung University College of Medicine, Taoyuan, Taiwan; c Hemodialysis Center, Taoyuan Chang Gung Memorial Hospital, Taoyuan, Taiwan; d Department of Pathology, Chang-Gung Memorial Hospital, Linkou, Taiwan.

**Keywords:** case report, glomerulocystic disease, young diabetes

## Abstract

**Rationale:**

This case report presents a challenging medical scenario involving a young adult male who exhibited an unusual combination of symptoms, including abrupt weight loss, declining renal function, proteinuria, and concurrent onset of diabetes mellitus. Remarkably, the patient had no previous medical history or family history of similar conditions, necessitating a comprehensive investigation.

**Patient Concerns:**

On March 10, 2021, a 25-year-old male sought medical attention due to the aforementioned symptoms. Initial assessments revealed stage 5 chronic kidney disease, with elevated blood urea nitrogen (BUN) and serum creatinine (Cr) levels, as well as significant proteinuria. The only notable physical finding was obesity, and renal ultrasound showed normal-sized kidneys without cysts.

**Diagnosis:**

A treatment plan was initiated to stabilize creatinine levels, including medications such as Glimepiride, Glyxambi, Bisoprolol, Amlodipine, and Valsartan. However, despite diligent medication management, proteinuria persisted, prompting further evaluation. A renal biopsy was performed on April 12th, 2023, leading to the diagnosis of glomerulocystic kidney disease with early-stage changes indicative of diabetic nephropathy.

**Interventions:**

The patient continues to receive ongoing care and follow-up at our outpatient clinic to optimize therapeutic interventions and elucidate the underlying etiology of this complex clinical scenario.

**Outcomes:**

Ongoing investigations and therapeutic interventions are crucial to understand the underlying cause and optimize patient care in this intricate clinical scenario.

**Lessons:**

This case underscores the complexity of diagnosing and managing a young adult presenting with concurrent renal dysfunction, proteinuria, and diabetes mellitus in the absence of prior underlying conditions. It highlights the importance of comprehensive evaluation and ongoing care in such challenging cases.

## 1. Introduction

Glomerulocystic kidney disease (GCKD) represents a less common form of cystic renal ailment, setting itself apart from the more widely recognized types of cystic renal diseases. Dating as far back as 1941, Roos documented a case involving an infant who displayed symptoms of failure to thrive, rickets, and renal failure, all linked to the presence of cystic glomeruli.^[1]^ A defining feature of this condition is the presence of cortical microcysts, which manifest as cystic dilatation of Bowman spaces. GCKD exhibits associations with various inherited diseases, including autosomal dominant polycystic kidney disease,^[2]^ tuberous sclerosis complex,^[3]^ medullary cystic kidney disease,^[4]^ nephronophthisis,^[5]^ Down syndrome,^[6]^ and maturity-onset diabetes of the young,^[7,8]^ among others. In this report, we present a young case characterized by deteriorating renal function and a recent diagnosis of diabetes mellitus, ultimately leading to the identification of GCKD through renal pathology.

## 2. Case presentation

We present the complex case of a young adult male who experienced an unusual constellation of symptoms, including abrupt weight loss, deteriorating renal function, proteinuria, and the concurrent onset of diabetes mellitus. Notably, the patient had no previous history of underlying diseases or a family history of similar conditions. On March 10, 2021, a 25-year-old male sought medical attention at our outpatient clinic due to the aforementioned symptoms. Initial laboratory investigations confirmed stage 5 of chronic kidney disease, with a blood urea nitrogen (BUN) level of 46.5 mg/dL and a serum creatinine (Cr) level of 5.72 mg/dL. Additionally, the patient exhibited substantial proteinuria, as evidenced by an urine albumin-to-creatinine ratio (alb/Cr) of 2306.7 mg/g and a urine total protein-to-creatinine ratio of 3444.5 mg/g. Physical examination revealed obesity (body mass index, BMI: 35.81) as the only significant finding. The renal ultrasound examination revealed kidneys of normal size without any discernible cysts.

**Figure 1.**
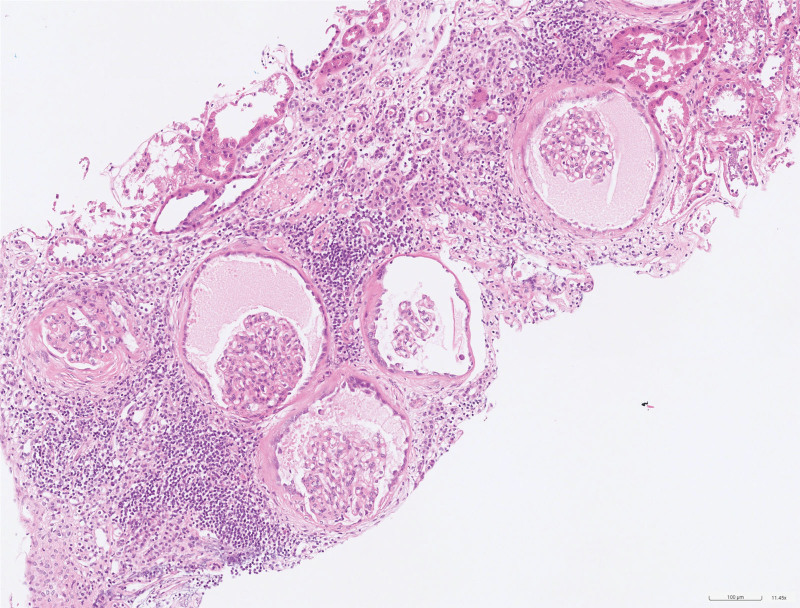
The biopsy reveals numerous glomeruli exhibiting cystically expanded Bowman space.

**Figure 2.**
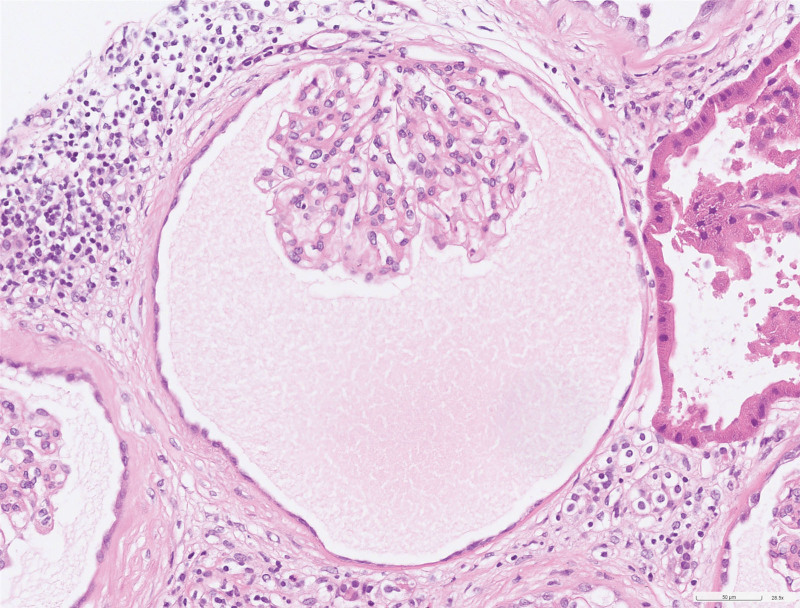
The native glomeruli are observed within the cysts.

A therapeutic regimen was initiated to stabilize the patient creatinine levels, including Glimepiride 2 mg once daily, Glyxambi 25/5 mg once daily, Bisoprolol 5 mg once daily, Amlodipine 5 mg once daily, and Valsartan 160 mg once daily. Despite diligent medication management, the progression of proteinuria persisted, prompting discussions and recommendations for further evaluation. Renal biopsy was performed on April 12th, 2023, following the patient admission on April 9th, 2023. Prior to the biopsy, a series of blood tests (Table 1) showed Hemoglobin (HB) 15.4 g/dL, BUN 33.2 mg/dL, Creatinine (Cr) 2.14 mg/dL, alanine transaminase 17 U/L, Albumin 4.4 mg/dL, Total Cholesterol (TC) 217 mg/dL, Triglycerides (TG) 436 mg/dL, HBsAg Nonreactive, Anti-hepatitis C virus Antibodies Nonreactive, immunofixation electrophoresis no monoclonal protein detected, Anti-double stranded DNA (Anti-dsDNA) Negative, Normal C3 and C4 levels, rapid plasma reagin nonreactive, anti-Streptolysin O Nonreactive, rheumatoid factor < 11.6 IU/mL, IgG 1070 mg/dL, IgA 232 mg/dL, IgM 96.9 mg/dL, IgD < 13.38 mg/L, IgE 203 mg/dL, myeloperoxidase-anti-neutrophil cytoplasmic antibodies negative, proteinase 3-anti-neutrophil cytoplasmic antibodies negative. Renal biopsy revealed glomerulocystic kidney disease (Figs. 1 and 2), and no complications were observed post-biopsy. Furthermore, electron microscopy analysis revealed the following details: Within one glomerulus, there was evidence of segmental mesangial nodular expansion, along with a segmental, mild thickening of the glomerular capillary loop basement membrane, measuring approximately 600 to 700 nanometers in thickness. Notably, electron-dense material was observed in the paramesangial area. Based on its morphology, it is more likely to be serum exudate rather than deposits of immune complexes. Importantly, no diffuse podocyte foot process effacement was detected. These findings indicate early-stage changes characteristic of diabetic nephropathy.

**Table 1:** Blood/urine tests before renal biopsy.

Variables	Value	Normal range (unit)
Hb	15.4	13.5–17.5 g/dL
WBC	8.9	3.9–10.6 X1000/uL
BUN	33.2	6–21 mg/dL
Cr	2.14	M:0.64~1.27, F:0.44~1.03 mg/dL
Urinary Alb/Cr*	1855.2	<30 mg/g
Urinary TP/Cr*	2437.3	<150 mg/g
ALT	17	<36 U/L
Serum Albumin	4.4	3.5–5.2 g/dL
TC	217	<200 mg/dL
TG	436	<150 mg/dL
HBsAg	Nonreactive (0.36)	Nonreactive < 0.9 COI
Anti-HCV antibody	Nonreactive (0.034)	Nonreactive < 0.9 COI
Serum IFE	No monoclonal protein detected	No paraprotein is identified
Anti-dsDNA	Negative (11.7)	Neg ≦ 92.6, Eq: 92.7~138.9, *P*: >139 unit/mL
C3 levels	128.2	90–180 mg/dL
C4 levels	25.4	10–40 mg/dL
RPR	Nonreactive	Nonreactive
ASLO	Nonreactive (60.1)	< 200 IU/mL
RF	< 11.6	< 15 IU/mL
IgG	1070	700–1600 mg/dL
IgA	232	70–400 mg/dL
IgM	96.9	40–230 mg/dL
IgD	< 13.38	<132.1 mg/L
IgE	203	<100 IU/mL
MPO-ANCA	Negative (0.2)	Neg < 3.5, Eqi: 3.5–5, Pos > 5
PR3-ANCA	Negative (0.2)	Neg < 2, Eqi:2–3, Pos > 3

Alb/Cr = albumin-to-creatinine ratio, ALT = alanine transaminase, Anti-dsDNA = anti-double stranded DNA, ASLO = anti-streptolysin O, BUN = blood urea nitrogen, Cr = creatinine, Hb = hemoglobin, HBsAg = hepatitis B surface antigen, HCV = hepatitis C virus, IFE = immunofixation electrophoresis, MPO-ANCA = myeloperoxidase-anti-neutrophil cytoplasmic antibodies, PR3-ANCA = proteinase 3-anti-neutrophil cytoplasmic antibodies, RF = rheumatoid factor, RPR = rapid plasma reagin, TC = total cholesterol, TG = triglycerides, TP/Cr = total protein-to-creatinine ratio, WBC = white blood cell counts.

* Represents urine sample.

The patient continues to receive ongoing care and follow-up at our hospital outpatient clinic.

## 3. Discussion

We present a case of a youthful individual experiencing newly diagnosed diabetes mellitus (DM), renal failure, and concomitant complications attributed to glomerulocystic kidney disease (GCKD). GCKD is an uncommon medical condition that was first described in 1941 by Roos, who identified an isolated abnormality in the kidneys.^[1]^ The terminology “glomerulocystic kidney disease” was coined by Taxy and Filmer to characterize the dilation of Bowman space.^[9]^ While the exact underlying mechanism remains poorly understood, histological examination has revealed the presence of dilated or enlarged Bowman capsules alongside glomerular cysts and normal tubules.

GCKD can be classified into 5 primary categories^[10]^:

1. Familial non-syndromic.
2. Associated with inheritable malformation syndromes.
3. Syndromic, non-mendelian.
4. Sporadic.
5. Acquired and dysplastic kidneys.

Interestingly, a subset of GCKD cases, categorized under group 5, has been observed primarily in individuals with a history of hemolytic-uremic syndrome.^[11]^ GCKD displays distinct clinical presentations categorized as early-onset (more common in neonates) and late-onset (more common in adults).^[12]^ In early-onset GCKD, renal function remains stable for several years, with the potential to progress to end-stage renal disease within a span of 3 or more years. In contrast, late-onset GCKD tends to exhibit milder renal impairments. Thankfully, the development of end-stage kidney disease due to GCKD is uncommon during childhood.^[13]^

The varying clinical profiles observed in GCKD may be attributed to superimposed glomerulonephritis,^[14]^ age or other distinct underlying conditions, such as diabetes mellitus.^[9]^

Imaging studies, such as computed tomography (CT) and magnetic resonance imaging (MRI) scans, are essential for diagnosing GCKD. These images typically reveal the presence of small renal cysts that exhibit a predominant distribution in the cortical and subcapsular regions.^[15]^ It is worth noting that when compared to other cystic kidney diseases, conventional renal ultrasound may not provide sufficient information for differentiation. In our patient, because of impaired renal function, we opt for renal ultrasound rather than computed tomography with contrast agent injection for imaging examinations.

Up to today, research on the relationship between early-onset diabetes and GCKD has advanced into the field of genetics. Mutations in the gene encoding hepatocyte nuclear factor (HNF)-1β are associated with early-onset diabetes and renal cystic disease.^[16]^ HNF-1β plays a pivotal role in the intricate process of embryonic renal development, contributing significantly to the branching of the ureteric bud and the formation of nephrons. Beyond its critical function in kidney development, HNF-1β also serves as a transcription factor with broad implications, regulating gene expression in the epithelial cells of several other vital organs, including the pancreas, the paramesonephric duct and liver.^[17,18]^ This condition exhibits a broad clinical spectrum of renal developmental anomalies, encompassing renal cysts, GCKD, and solitary kidneys. Additionally, it is associated with systemic manifestations like diabetes mellitus, gout, and malformations in the genital tract.^[19]^

In summary, GCKD is a rare condition characterized by the expansion of Bowman space. It can be classified into 5 main categories, depending on hereditary, syndromic, or acquired factors. Achieving an accurate diagnosis often necessitates advanced imaging modalities such as CT, MRI, and renal biopsy due to the distinctive distribution pattern of renal cysts found in GCKD.

## 4. Conclusion

This case underscores the complexity of diagnosing and managing a young adult presenting with concurrent renal dysfunction, proteinuria, and diabetes mellitus in the absence of prior underlying conditions. Despite initial treatment to stabilize renal function, proteinuria persisted, prompting the need for a renal biopsy, which ultimately revealed glomerulocystic kidney disease. Ongoing investigations and therapeutic interventions remain crucial in elucidating the underlying etiology and optimizing patient care in such intricate clinical scenarios.

## Author contributions

**Conceptualization:** Wen-Hung Huang, Kun-Hua Tu.

**Data curation:** Wen-Hung Huang, Kun-Hua Tu, Tai-Di Chen.

**Investigation:** Cheng-Hao Weng.

**Methodology:** Tai-Di Chen.

**Supervision:** Wen-Hung Huang, Ching-Wei Hsu.

**Writing – original draft:** Wen-Hung Huang, Kun-Hua Tu.

**Writing – review & editing:** Cheng-Hao Weng, Ching-Wei Hsu.
